# A new fluorescent dye accumulation assay for parallel measurements of the ABCG2, ABCB1 and ABCC1 multidrug transporter functions

**DOI:** 10.1371/journal.pone.0190629

**Published:** 2018-01-17

**Authors:** Edit Szabó, Dóra Türk, Ágnes Telbisz, Nóra Kucsma, Tamás Horváth, Gergely Szakács, László Homolya, Balázs Sarkadi, György Várady

**Affiliations:** 1 Institute of Enzymology, Research Centre for Natural Sciences, Hungarian Academy of Sciences, Budapest, Hungary; 2 Department of Experimental Pharmacology, National Institute of Oncology, Budapest, Hungary; 3 Institute of Cancer Research, Medical University Vienna, Vienna, Austria; 4 Department of Biophysics and Radiation Biology, Semmelweis University, Budapest, Hungary; Columbia University, UNITED STATES

## Abstract

ABC multidrug transporters are key players in cancer multidrug resistance and in general xenobiotic elimination, thus their functional assays provide important tools for research and diagnostic applications. In this study we have examined the potential interactions of three key human ABC multidrug transporters with PhenGreen diacetate (PGD), a cell permeable fluorescent metal ion indicator. The non-fluorescent, hydrophobic PGD rapidly enters the cells and, after cleavage by cellular esterases, in the absence of quenching metal ions, PhenGreen (PG) becomes highly fluorescent. We found that in cells expressing functional ABCG2, ABCB1, or ABCC1 transporters, cellular PG fluorescence is strongly reduced. This fluorescence signal in the presence of specific transporter inhibitors is increased to the fluorescence levels in the control cells. Thus the PG accumulation assay is a new, unique tool for the parallel determination of the function of the ABCG2, ABCB1, and ABCC1 multidrug transporters. Since PG has very low cellular toxicity, the PG accumulation assay also allows the selection, separation and culturing of selected cell populations expressing either of these transporters.

## Introduction

Several members of the ATP-binding cassette (ABC) superfamily of membrane transporters are working as efflux pumps for a large variety of xenobiotics and drugs. Therefore, these transporters are important players in multidrug resistance against anti-cancer therapeutic compounds, and also significantly modify the absorption, distribution, metabolism, excretion and toxicity (ADME-Tox) parameters for numerous therapeutic agents. The three key ABC efflux transporters involved in human cancer drug resistance and drug metabolism are the ABCB1 (P-glycoprotein, Pgp), the ABCC1 (multidrug resistance protein 1, MRP1) and the ABCG2 (breast cancer resistance protein, BCRP) proteins, thus their evaluation has a major importance in drug development and clinical diagnostics [1–6]. Due to the promiscuity of these proteins in drug binding and transport, the molecular mechanisms of drug interactions and the potential drug-drug interactions caused by the expression and function of these transporters are largely unexplored. Recent structural and modeling data for these ABC transporters [7,8] are still insufficient to predict substrate interactions at a molecular level, thus experimental techniques to assess these interactions are of utmost importance.

Data on ABC multidrug transporter protein expression and localization have to be complemented with efficient functional assays in order to evaluate the potential effects of transporters on drug interactions. There are various assays assessing the function of these ABC transporters, including drug-stimulated ATPase activity, direct drug transport measurements in whole cells or in inverted membrane vesicles, and a widely applied assay system is to follow the extrusion of fluorescent transporter substrates from living cells [9,10]. Transporter substrate dyes, becoming fluorescent when interacting with cellular DNA (e.g. Hoechst 33342 or DCV), have been efficiently used to study the cellular function of these transporters [10–14], but these compounds have long-term toxic effects and no such dye has been found as a common substrate for all the three major ABC drug transporters. A transporter assay was reported with protected by patent structures of the dyes, eFluxx-IDH Green and Gold, suggesting a parallel examination of the three multidrug transporters, although this dye has relatively high toxicity [15].

A significant amplification of the sensitivity of the cellular transporter assays is achieved when the substrate extruded by the ABC transporter is non-fluorescent, and the cellular metabolism-dependent accumulation of a highly fluorescent derivative is strongly reduced by the action of the transporter. Such an assay for ABCB1 and ABCC1, by using e.g. the non-toxic cell viability dye Calcein AM, is already available [16–18].

In this report we document that the application of PhenGreen SK diacetate (PGD) allows a parallel and sensitive functional detection of all these three major ABC multidrug transporters. PGD is a non-fluorescent, hydrophobic molecule, which rapidly enters the cells, where PGD is cleaved by non-specific esterases to yield a highly fluorescent hydrophilic dye, PhenGreen (PG), trapped inside the cell. The green fluorescence of PG is variably quenched in the presence of divalent metal ions, especially by heavy metal ions [19]. Therefore, this PGD loading and PG fluorescence measurement technology has been applied for the determination of iron or cadmium ions in various cellular systems [20–22].

Interestingly, as we show here, PG accumulation is strongly reduced by the function of the ABCG2, ABCB1, as well as by the ABCC1 transporter. We document that under appropriate assay conditions, in the absence of divalent quenching ions, fluorescent PG accumulation can be efficiently used for a functional assay of all these drug transporters. Flow cytometry and fluorescence microscopy, allowing high-throughput and high-content assays, are both suitable for performing these measurements, and short-term PG accumulation is non-toxic to the cells. Parallel application of selective transporter inhibitors make this assay a simple, versatile and sensitive tool to assess specific ABC multidrug transporter function.

## Materials and methods

### Materials

PhenGreen SK diacetate (PGD) was purchased from Thermo Fischer Scientific (Waltham, MA, US). KO143 was obtained from Tocris Bioscience (Bristol,UK). Benzbromarone and mitoxantrone were purchased from Sigma-Aldrich-Merck (St. Louis, USA). Tariquidar was a kind gift from Dr. S. Bates (NCI, NIH). The 5D3 antibody was purified from ABCG2-5D3 hybridoma cell line (a kind of gift of Dr. Brian Sorrentino). QCRL3 with labeled AlexaFluor 488 antibody was obtained from Sony Biotechnology (Surrey, UK). MRK16 antibody was obtained from Kamiya Biomedical Company (Seattle, US). Bxp-21 was purchased from Abcam (Cambridge, UK). C219 antibody was obtained from Enzo Life Sciences (New York, USA). The secondary antibodies (AlexaFluor 488 and 647), Wheat Germ Agglutinin (WGA)-AlexaFluor 647 and TO-PRO™-3 Iodide were purchased from Thermo Fischer Scientific (Waltham, MA, US). Calcein AM (Ca-AM) was obtained from Thermo Fischer Scientific (Waltham, MA, US). Components of phosphate buffered saline were obtained VWR (Radnor, Pennsylvania, USA). All other materials, if unless otherwise were obtained from Sigma-Aldrich-Merck (St. Louis, USA).

### Cell lines

PLB-985 myelomonocytic and A431 skin derived epidermoid carcinoma cell lines, stably expressing the ABCG2 or the ABCB1 protein were generated by using a retroviral transduction system [23–25]. HEK-293 human embryonic kidney and HL-60 human promyelocytic leukemia cell lines stably expressing ABCC1 were also generated by retroviral transduction [26]. Stable expression of the ABC multidrug transporters in these cell lines was regularly examined by specific immunostaining and flow cytometry analysis, using the MRK-16 and C219 (ABCB1), QCRL3 and MRPm6 (ABCC1) and 5D3 and Bxp-21 (ABCG2) antibodies, respectively (see **S1–S3 Figs**).

The cellular esterase activity of PLB-985 and HL-60 cell lines was measured by the Calcein assay. The control, and ABCG2, ABCB1 or ABCC1 transporter expressing cells were incubated with specific transporter inhibitors (ABCG2 by 2.5μM KO143 (KO), ABCB1 by 0.25μM tariquidar (TQ), and ABCC1 by 50μM benzbromarone (BB)). The cells were incubated with 0.1 μM or 0.5μM Calcein AM in DPBS for 1–15 minutes at 37°C. Dye uptake was stopped by the addition of 150μl ice-cold DPBS, the cells were kept on ice until the measurements (see **S4 Fig**).

### Flow cytometry

Immunostaining and the transport activity of the ABC transporters were measured by FacsCanto II flow cytometer (BD Bioscience, San Jose, CA) equipped with a blue (488 nm) and red (633 nm) lasers. The PhenGreen (PG) or the Calcein signal was detected in the FITC channel (emission filter: 530/30 nm), mitoxantrone (MX) and TO-PRO-3 signals were detected in the APC channel (emission filter: 660/20 nm).

### PhenGreen accumulation measurements by flow cytometry

In order to follow the time-dependent accumulation of PG, 5×10^5^ cells were washed twice with 1mL DPBS (1g/L D-glucose with phosphate buffered saline), then pre-incubated in the uptake buffer (1mM EDTA in DPBS) for 10 minutes at 37°C. Thereafter the cells were incubated in the uptake buffer with various concentrations of PhenGreen SK diacetate (PGD), with or without transporter inhibitor, at 37°C, for 1–60 minutes. Dye uptake was stopped by the addition of 150μl ice-cold EDTA-DPBS, the cells were kept on ice until the measurements (see **S5 Fig**). For assessing the PGD concentration dependence of PG accumulation, 5×10^5^ PLB-985 or A431 cells, expressing the ABCB1 or ABCG2 transporters, or HL-60 and HEK-293 cells, expressing ABCC1, were washed twice with 1mL DPBS, then pre-incubated in EDTA-DPBS medium for 10 minutes at room temperature. The cells were incubated in EDTA-DPBS with 0.1–5μM of PGD at 37°C for 30 minutes. Dye uptake was stopped by the addition of 150μl ice-cold EDTA-DPBS. The cells were kept on ice until the measurement.

For assessing transporter inhibition, the ABCG2 transporter function was inhibited by 2.5μM KO, ABCB1 by 0.25μM TQ, and ABCC1 by 50μM BB. The cells (5×10^5^) were incubated with 0.5μM PGD or 1μM (Mx) with or without inhibitors, for 30 (PGD) or 60 (Mx) minutes at 37°C. The reaction was stopped by the addition of ice-cold EDTA-DPBS and fluorescence was measured as described above.

In order to the potential effects of metal ions in the cells or in the media, we have used EDTA both in the washing and incubation media. Without the use of this metal chelator we obtained variable results for the transporter activity, while the use of EDTA in all media allowed reproducible studies, and made it unlikely that cellular metal concentrations would affect the probe fluorescence or its transport properties.

The efflux of PhenGreen was measured by FACS Aria III Cell sorter (BD Bioscience, San Jose, CA, with 488nm blue laser excitation and 530/30 nm emission). The control, and ABCG2, ABCB1 or ABCC1 transporter expressing cells were treated with specific transporter inhibitors (ABCG2 by 2.5μM KO, ABCB1 by 0.25μM TQ, and ABCC1 by 50μM BB). The cells were incubated with 1μM PGD in EDTA-DPBS for 30 minutes at 37°C, then washed three times and the efflux measured continuously for 40 minutes at 37°C (see **S6 Fig**).

### Flow cytometry data analysis

All experiments were performed at least three times. Data analysis was performed using FACSDiva v6.1.3 Software (BD Bioscience, San Jose, CA), flow cytometry figures were prepared with the Attune Acoustic Focusing Cytometer v1.25 Software (Applied Biosystems, Life Technologies, Carlsbad, CA, USA). Results were expressed as median ± standard deviation. The MDR activity factor % (MAF%—see refs [17,26]) was calculated as follows: MAF% = (((MFI_inh_-MFI_0_)/MFI_inh_)×100), wherein MFI_inh_ and MFI_0_ are the median fluorescence intensity (MFI) with (inh) or without (0) inhibitor. The EC_50_ analysis was carried out using the Origin 8.6. software.

### Cell viability assay

Control and ABCG2 expressing PLB-985 cells (1×10^6^) were pre-incubated in the uptake buffer for 10 minutes at 37°C, then incubated with or without 0.5μM PGD for 30 minutes at 37°C. The PGD-treated cells were sorted based on PG fluorescence by FACS Aria III Cell sorter. The sorted cells were suspended in 3mL RMPI media in 6 well plates and live cell number was measured each day, dead cells were excluded by TO-PRO™-3 Iodide.

In order to estimate the EC_50_ values, HEK or A431 cells were treated with 0.5 μM PGD for 30 min at 37°C in the EDTA-DPBS buffer, then washed and cultured in 2 mL DMEM media in 12 well plates for 72 hours. Live cell number was determined by FACSCantoII flow cytometer, dead cells were excluded by TO-PRO™-3 Iodide.

### Confocal images

For confocal microscopy the cells (5×10^5^) were washed twice with 1mL DPBS, then pre-incubated with 1μg/mL Alexa Fluor-647 conjugated wheat-germ agglutinin (WGA-A647) in uptake buffer for 5 minutes at room temperature. Thereafter the cells were incubated with 0.5μM PGD with or without transporter inhibitors for 30 minutes at 37°C. PGD uptake was stopped by washing the cells with 1mL DPBS. The images were acquired by a Zeiss LSCM 710 microscope using a 63×NA = 1.4 Plan Apo objective. Images were captured and analyzed by Zen2 (Blue edition) Software.

## Results and discussion

### Cell lines and assay conditions

In this study we examined the potential interactions of three key multidrug transporters, ABCG2 (BCRP), ABCB1 (MDR1, Pgp), and ABCC1 (MRP1) with a compound, originally applied for detecting intracellular metal ion concentrations. PhenGreen diacetate (PGD) is a hydrophobic, cell permeable molecule, which inside the cells is cleaved by cellular esterases into fluorescent PhenGreen (PG), and this hydrophilic product is accumulated inside the cells. Interaction of PG with various metal ions results in the quenching of PG fluorescence, thus allows the quantitative estimation of cellular metal concentrations [20–22].

In order to investigate the interactions of the ABC transporters with PGD, we used various cell lines expressing ABC transporters. As described in the methods section, and documented in detail in the **S1–S3 Figs**, the stable and selective overexpression of the respective transporters was assured by continuous examination of the transporter levels by immunostaining in flow cytometry and Western blotting. The cell lines applied in this study included the PLB/HL-60 lymphoblastoid cells, selectively overexpressing ABCG2, ABCB1 or ABCC1, the A431 cells overexpressing ABCG2 or ABCB1, and the HEK cells, overexpressing ABCC1. In each case the respective control cells, with unmeasurably low expression levels of these transporters, were also applied (**S1–S3 Figs**).

In order to select appropriate conditions for studying the effects of ABC transporters on cellular PG accumulation, we examined the rate of PGD uptake and PG accumulation in the applied cell lines, at various PGD concentrations (between 0.5–2.5μM) in the media. In these studies, we used metal-free media (see Methods) in order to exclude quenching of the PG fluorescence. Based on several trial conditions, as an optimal, non-toxic media for the cell based assays we used the glucose-containing DPBS, supplemented with 1mM EDTA.

As shown in a time-course experiment documented in **S5 Fig**, in the PLB cells, when 0.5 to 2.5μM PGD was applied in the media, the increase in cellular PG fluorescence at 37°C saturated in less than 30 minutes. Also, under these conditions the effects of the ABC transporters (shown for ABCG2 in **S5 Fig**), could be well assessed.

### Effects of ABCG2 on fluorescent PG accumulation

**Fig 1:**
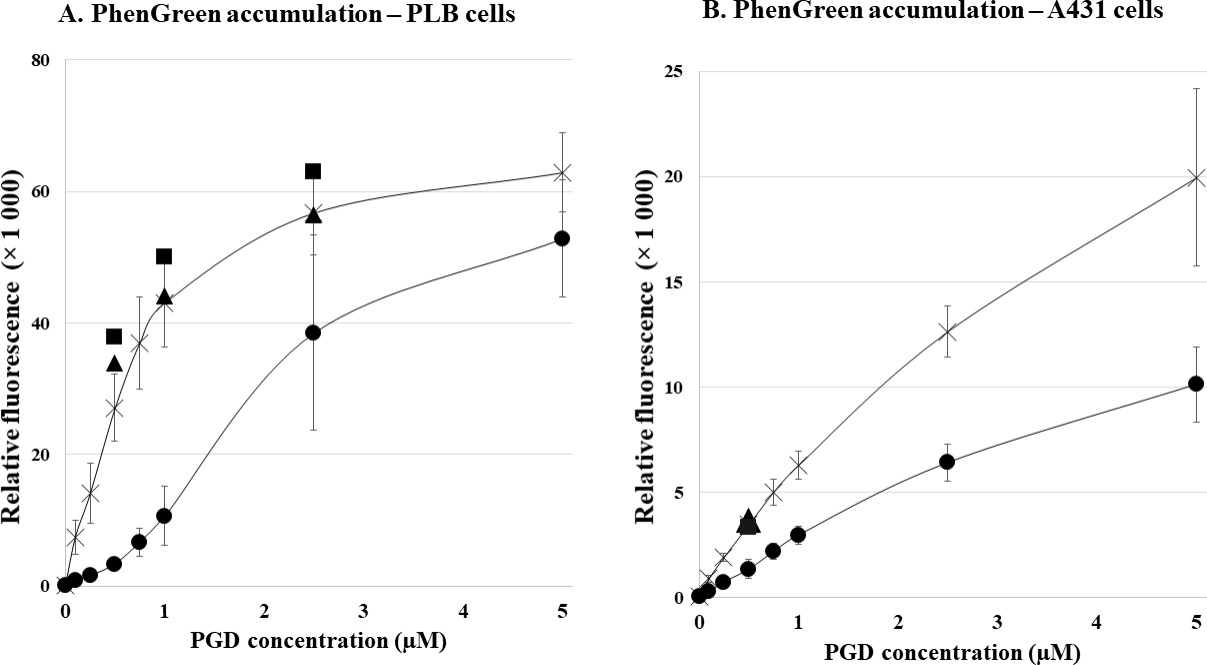
Fluorescent PG accumulation in human cells, effect of ABCG2 expression—Flow cytometry studies. **Panel A**. PGD-concentration dependent PG accumulation in control PLB cells (×) and in PLB-ABCG2 cells (●). The black squares (■: ABCG2) and triangles (▲: CTRL) demonstrate PG accumulation in the presence of 2.5μM KO143, a specific ABCG2 inhibitor. **Panel B.** PGD-concentration dependent PG accumulation in control A431 (×) and A431-ABCG2 (●) cells, measured in EDTA-DPBS medium for 30 minutes at 37°C. The black squares (■: ABCG2) and triangles (▲: CTRL) demonstrate PG accumulation in the presence of 2.5μM KO143 at 0.5μM PGD. ± SD values are indicated.

In the first set of experiments we analyzed in detail the effects of the cellular ABCG2 multidrug transporter expression on PG accumulation. **Fig 1A** shows the PGD-concentration dependence of PG accumulation in control PLB cells and in PLB-ABCG2 cells, respectively, as measured by flow cytometry. **Fig 1B** shows in similar experiments the PGD-concentration dependence of PG accumulation in control A431 and in A431-ABCG2 cells. As documented, in the PLB cells at low (below 1μM) PGD concentrations the presence of the ABCG2 protein in the cell membrane causes a major difference in the amount of the accumulated PG fluorescence, and this difference becomes somewhat smaller at higher PGD concentrations. In the case of the A431 cells, at increasing PGD concentrations the difference in cellular fluorescence caused by the ABCG2 protein still increases, while the ratio of the fluorescence values in the absence and presence of ABCG2, respectively, does not increase. Therefore, in our further experiments we used a PGD concentration of 0.5μM, which was found sufficient to provide optimum functional assay conditions.

In the following experiments we have studied the effects of ABCG2 variants and mutations of the cellular PG accumulation, and compared these effects with those on cellular mitoxantrone (MX) accumulation. MX is a well-established fluorescent transported substrate of the ABCG2 protein, and this drug is widely used to assess ABCG2 function. However, MX is a strongly cytotoxic agent and cannot be used for separation or further culturing of transporter expressing cells.

**Fig 2:**
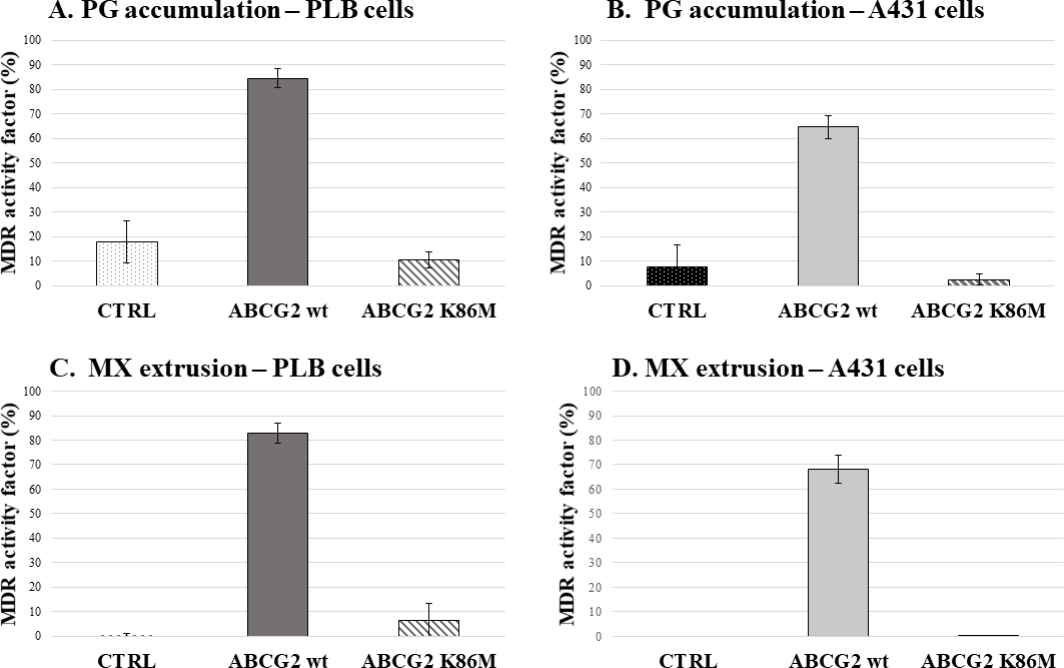
MDR activity factors based on PG and mitoxantrone (MX) accumulation in human PLB and A431 cells. **Effects of the ABCG2 variants on dye extrusion capacity—flow cytometry studies. Panels A. and B.**: MDR activity factor (see Methods) calculated by PG accumulation in PLB cells (Panel A) and A431 cells (Panel B), expressing ABCG2 variants. Panels C. and D.: MDR activity factor calculated by MX accumulation in PLB cells (Panel C) and A431 cells (Panel D), expressing ABCG2 variants. +/- SD values are indicated.

In the experiments shown in **Fig 2**, we present the MDR activity factors (see refs [17,26] calculated from the differences in the fluorescent cellular PG accumulation, found in the absence or the presence of a specific inhibitor of ABCG2, Ko143. As shown in Panel A, control PLB cells have a very low level of MDR activity, while PLB cells expressing the wild-type (wt) ABCG2 protein show a high activity level. In order to demonstrate the role of ABCG2 transporter activity in this process, we also examined the effect of the non-functional catalytic mutant ABCG2-K86M on PG accumulation. As expected, this mutant variant, also with somewhat lower membrane expression (see **S1 Fig**) had no effect on PG accumulation, thus produced a low MDR activity factor. Panel B documents similar PG accumulation studies in control and ABCG2 expressing A431 cells, respectively, with essentially the same findings in this cell line.

In parallel experiments we have also calculated the MDR activity factor based on MX extrusion, in the control and ABCG2 expressing PLB and A431 cells, respectively. As shown in Panels C and D in **Fig 2**, MX extrusion measurements gave essentially similar results as obtained by using PG accumulation.

**Fig 3:**
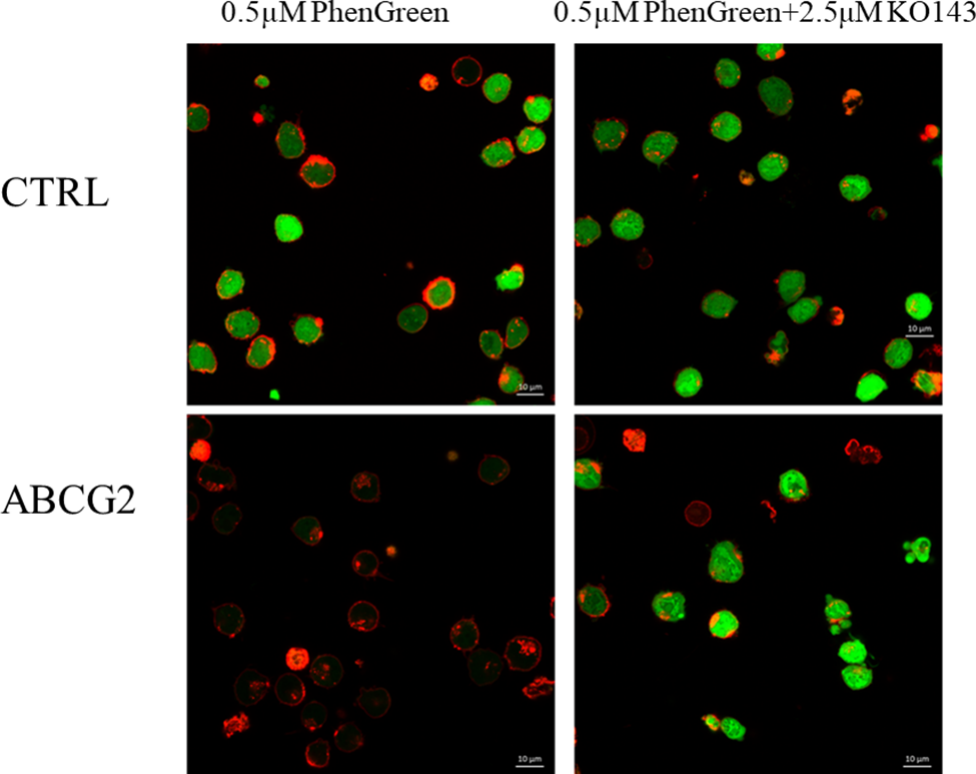
Fluorescent PG accumulation in human PLB cells, examined by confocal microscopy. **Effects of ABCG2 protein expression and the specific inhibition of ABCG2 function by Ko143.** Cellular fluorescence was observed by confocal microscopy. PG fluorescence (green) was examined after 30 minutes of the addition of 0.5μM PGD to the medium, either in the absence or presence of the ABCG2 inhibitor KO143 (2.5μM). The cells were pre-labeled with fluorescent anti-WGA (red) to indicate the plasma membranes.

In order to examine the wider applicability of the PGD-based assay for functional ABCG2 transporter studies, we examined PG accumulation in control PLB cells and in PLB cells expressing ABCG2, by using fluorescence microscopy. In these experiments, in order to label the plasma membrane compartment of the cells, we also included the staining of live PLB cells with a fluorescent anti-WGA antibody. As shown in the representative confocal microscopy images in **Fig 3**, control PLB cells showed an intensive green signal due to the cytoplasmic accumulation of PG, while the PLB-ABCG2 cells practically did not accumulate PG. Upon the addition of the specific ABCG2 inhibitor, Ko143, cellular fluorescence in the ABCG2 expressing cells was greatly increased.

**Fig 4:**
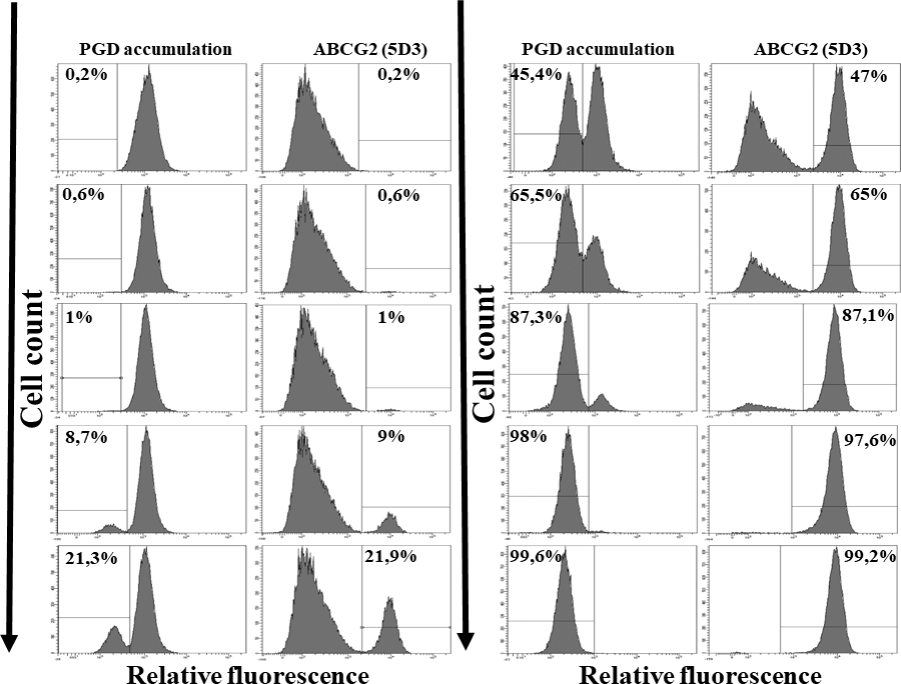
Flow cytometry detection of PG accumulation in human PLB cells—Recognition and separation of control PLB cells and PLB cells expressing the ABCG2 transporter. Control PLB cells and PLB cells expressing wild-type ABCG2 were mixed in various ratios (0.2–99.8%). PG accumulation was measured after the addition of 0.25μM PGD. Immunofluorescent detection of the ABCG2 protein on the cell surface of the same cells was measured by the ABCG2-specific 5D3 monoclonal antibody binding. The numbers on the graphs indicate the % values of the separated cells, measured based on the relative fluorescence values (see Methods).

In the following experiments we examined the potential use of the PG accumulation assay for the selection of cells expressing the ABCG2 protein. In case of tissue-derived or tumor stem cells the expression of the ABCG2 protein causes the appearance of a Side Population (SP), originally observed based on the Hoechst 33342 dye extrusion, due to the function of the ABCG2 protein (refs.[11,12,27–30]). As documented in **Fig 4**, based on low PG accumulation and its increase by the ABCG2 inhibitor KO143, even cell populations representing less than 1% of the total cell mixture, can be visualized and separated by using flow cytometry. This functional assay is similarly highly sensitive as the cell surface labeling of the ABCG2 protein by a specific monoclonal antibody, 5D3 (see **Fig 4**).

### Effects of ABCB1 and ABCC1 on fluorescent PG accumulation

In the case of the ABCB1 and ABCC1 multidrug transporters there are several fluorescence-based transporter assays available to estimate the function of these proteins. The DNA binding dyes, Hoechst 33342, MX and DCV are transported substrates of ABCB1, while these dyes are relatively poorly transported substrates of the ABCC1 protein. Acetoxymethyl esters of several fluorescent indicator dyes are transported by both ABCB1 [16], and Calcein AM, a non-toxic cell viability dye, which is actively extruded by both ABCB1 and ABCC1[26], is widely used for functional studies of these proteins. The transporter-dependent reduced accumulation of free Calcein, generated by cytoplasmic esterases, is a sensitive functional assay for both ABCB1 and ABCC1. In contrast, Calcein AM is not transported by ABCG2, thus this assay system cannot be used in the case of this transporter.

As shown above, the PG accumulation assay provides a sensitive assay for ABCG2 activity, therefore in the following experiments we examined if PG accumulation can also be applied to study the function of ABCB1 and/or ABCC1.

**Fig 5:**
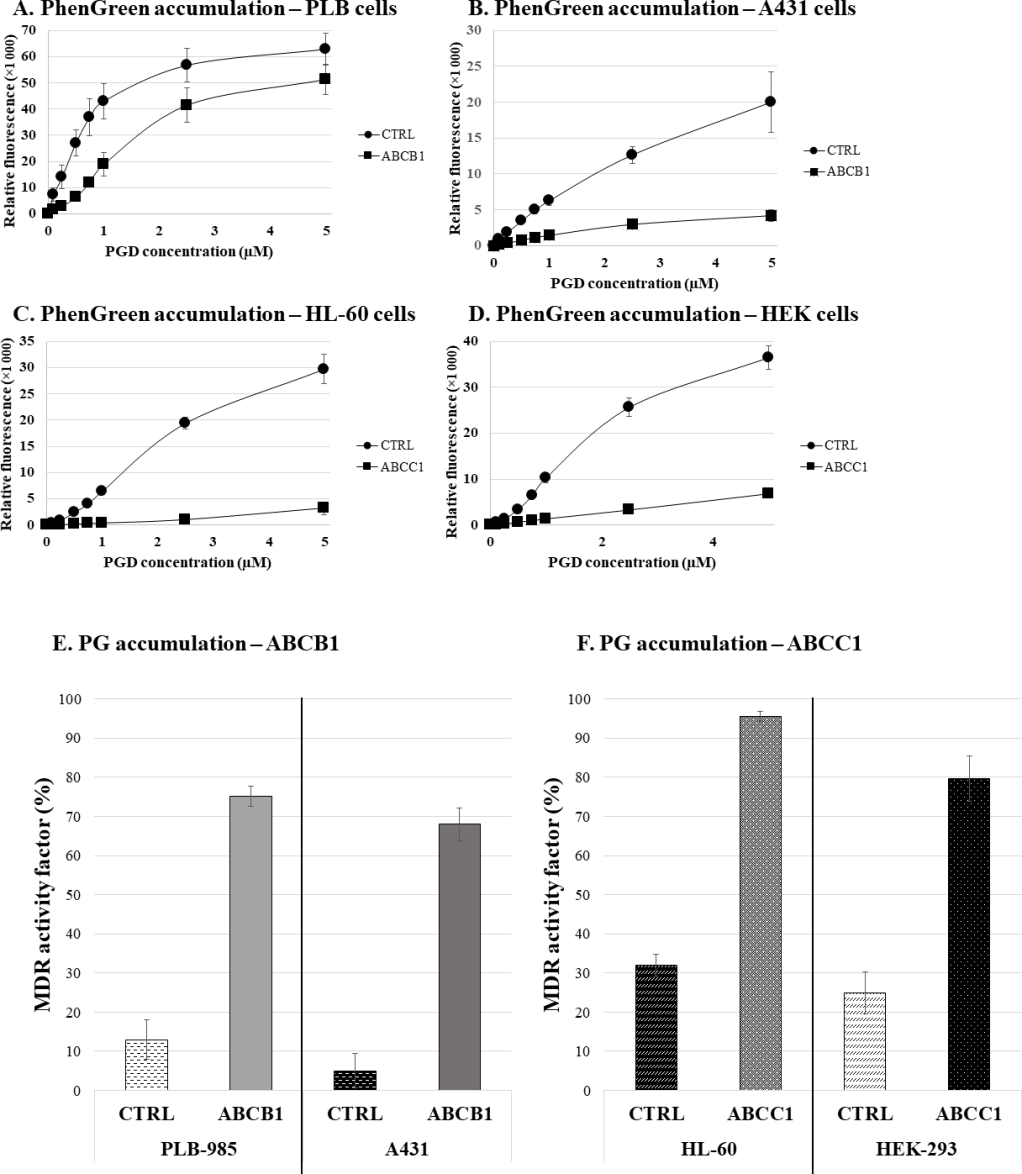
Fluorescent PG accumulation in human cells, effects of ABCB1 and ABCC1 expression—Flow cytometry studies. **Panel A**. Control PLB cells and ABCB1-expressing PLB cells, **Panel B**. Control A431 cells and ABCB1-expressing A431 cells, **Panel C.** Control HL-60 cells and ABCC1-expressing HL-60 cells, **Panel D.** Control HEK cells and ABCC1-expressing HEK cells. **Panel E**. Control PLB cells and ABCB1-expressing PLB cells, Control A431 cells and ABCB1-expressing A431 cells. **Panel F**. Control HL-60 cells and ABCC1-expressing HL-60 cells, Control HEK cells and ABCC1-expressing HEK cells.

As shown in **Fig 5**, in various human cell lines, expressing either ABCB1 or ABCC1, PG accumulation is significantly decreased. In a relatively wide concentration range, that is between 0.1–5μM, ABCB1 or ABCC1 expressing cells accumulate significantly lower levels of PG than their control parental cells. As shown in **Fig 5 Panel E**, the MDR activity factor can be calculated both by comparing the parental and transporter expressing cells, or by using selective inhibitors of ABCB1 (tariquidar) or ABCC1 (benzbromarone).

The cells were incubated in the presence of variable PGD concentrations (0.1–5μM) in EDTA-DPBS medium for 30 minutes at 37°C, and fluorescent PG accumulation in the cells was measured by flow cytometry.

In order to compare the efficiency of PG accumulation to determine the MDR activity factor, as compared to that when using mitoxantrone (MX) we have performed parallel experiments by using these two systems in ABCB1 and ABCC1 expressing cell lines, respectively. As documented in **S7 Fig**, the PGD assay as similarly or better applicable than MX for calculating the MDR activity factors for both of these transporter proteins.

We have also examined whether the potential differences in the cellular esterase activity in the different cell lines without or with ABC transporter expression may contribute to the found differences in PG accumulation. For this purpose, we applied Calcein AM, which is not a substrate of the ABCG2 transporter, and the same compound together with transporter inhibitors for ABCB1 or ABCC1. As shown in **S4 Fig**, the time course of the free Calcein accumulation, thus the cellular esterase activity, was the same in the parental and the ABCG2 expressing cells. These experiments have been repeated with ABCB1-expressing cells, by adding a specific inhibitor of the ABCB1 to both the control and the ABCB1-PLB cells. In all cases the esterase activity was very similar. In the case of ABCC1 expressing HL-60 cells the applied inhibitor, benzbromarone had a slight inhibitory effect on the esterase activity both in the control and the ABCC1-expressing cells, but the esterase activities were similar. These experiments indicate that the esterase activities are similar in the cells applied and the ABC transporters are responsible for the differences in PG accumulation.

**Fig 6:**
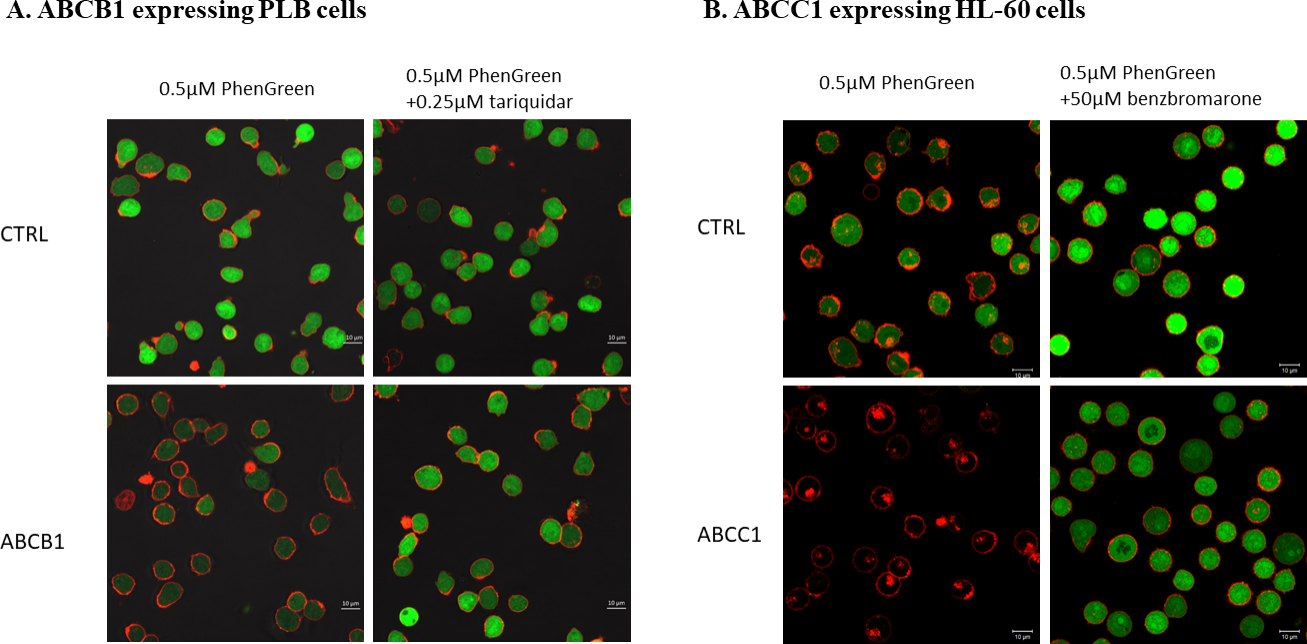
Fluorescent PG accumulation in human PLB and HL-60 cells, examined by confocal microscopy. **Effects of ABCB1 and ABCC1 protein expression and the specific inhibition of the transporter function by tariquidar (ABCB1) or by benzbromarone (ABCC1)**. Cellular fluorescence was observed by confocal microscopy. PG fluorescence (green) was examined after 30 minutes of the addition of 0.5μM PGD to the medium, either in the absence or presence of the transporter inhibitors (0.25μM tariquidar for ABCB1 or 50μM benzbromarone for ABCC1). The cells were pre-labeled with fluorescent anti-WGA (red) to indicate the plasma membranes.

In experiments directly following the cellular fluorescence by confocal microscopy (**Fig 6**), we have also documented the applicability of the PG accumulation assay to follow the activity of the ABCB1 and the ABCC1 multidrug transporters, respectively.

### Effect of PG accumulation on cell viability

When applying PGD treatment and PG accumulation for separation of cells variably expressing specific ABC multidrug transporters, an important point is the potential toxicity of the accumulated PG. Therefore, in the following experiments we have studied if PG accumulation has an effect on cell viability and cell growth.

PGD uptake and PG accumulation cannot be measured in cell culture media, as serum non-specific esterases rapidly cleave PGD to PG, and also, the presence of metal ions significantly change PG fluorescence. Therefore, a direct estimate of PGD cytotoxicity in cell cultures could not be performed, while the relevant, potential cellular effects of PGD and PG were estimated after the experimental period of PG loading.

**Fig 7:**
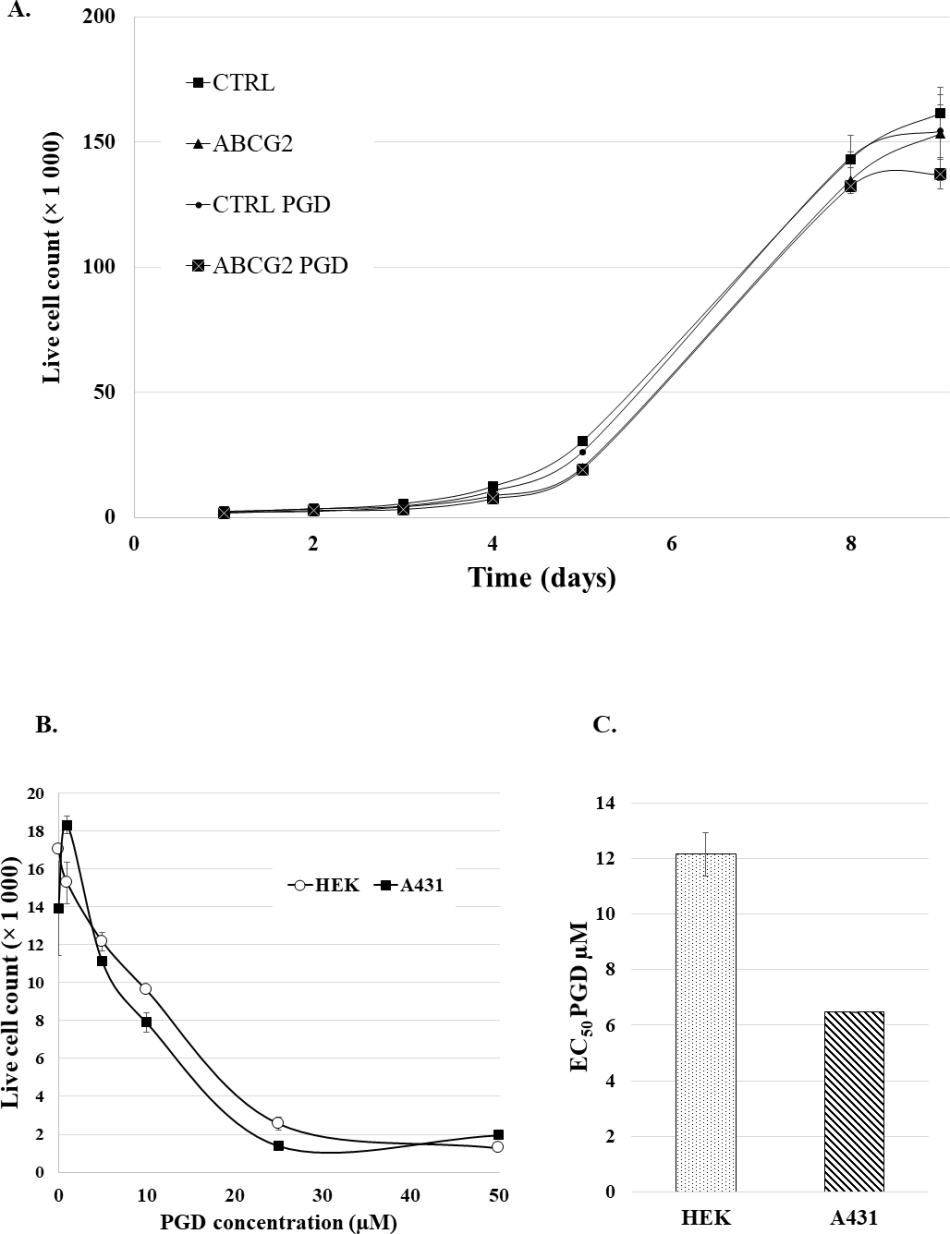
PhenGreen diacetate toxicity assay. **Panel A.** Effect of PhenGreen accumulation on cell growth in PLB cells and PLB-ABCG2 cell. Cell growth was measured after 0.5μM PGD treatment for 30 minutes at 37°C, followed by cell sorting. **Panels B and C.** Cytotoxic effects of PGD treatment in HEK and A431 cells. The cells were pre-treated with the indicated concentrations of PGD for 30 min at 37°C in the loading media, then washed and cultured in normal cell culturing media (see Methods) for 72 hours.

In the first set of these experiments (**Fig 7, Panel A**) we used control and ABCG2 expressing PLB cells, incubated in the PGD loading media with or without 0.5μM PGD for 30 minutes at 37°C. The PGD-treated cells were then sorted based on PG fluorescence by flow cytometry, and the cells were resuspended in RPMI media. Cell growth was estimated by cell counting for 9 days, and dead cells were excluded by TO-PRO-3 staining. In the second set of experiments HEK or A431 cells were treated with the indicated concentrations of PGD for 30 min at 37°C in the loading media, then washed and cultured in normal cell culturing media for 72 hours. Live cell number was determined by flow cytometry, dead cells excluded by TO-PRO-3 staining (see Methods). Based on these experiments, the PG accumulation used above for transporter activity studies and cell sorting had no measurable effect on the growth of the cells.

## Conclusions

**Fig 8:**
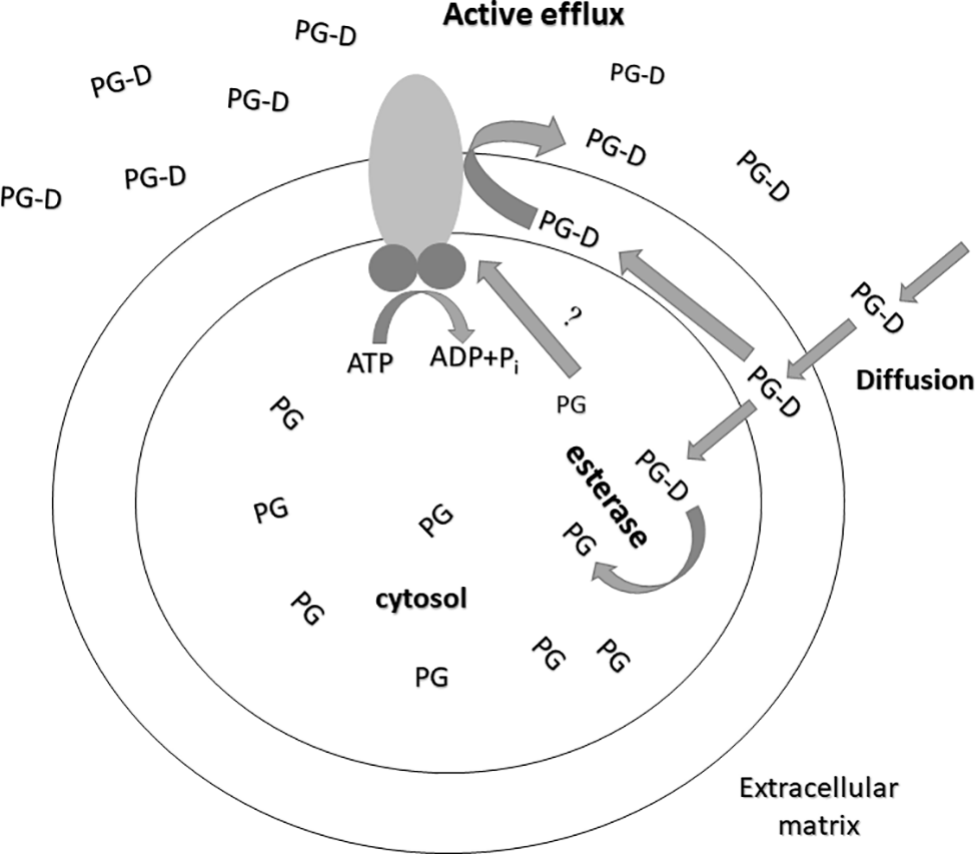
PhenGreen diacetate based assay for functional studies of multidrug resistance ABC transporters. The non-fluorescent, hydrophobic PGD rapidly enters the cells through the plasma membrane. In the cytoplasm, PGD is cleaved by nonspecific esterases to yield fluorescent Phengreen (*PG—for the structures of PGD and PG see **S8 Fig** [19]). The ABC transporters ABCG2, ABCB1, or ABCC1 efficiently extrude PGD (and potentially also PG) to the extracellular space.

In this study we have shown that in various human cells the expression of the multidrug transporters ABCG2, ABCB1 or ABCC1 strongly reduce fluorescent PG accumulation when the cells are incubated with PhenGreen SK diacetate (PGD). When using optimum assay conditions, that is metal ion free media and low PGD concentrations, ABC transporter function can be sensitively followed either by flow cytometry or fluorescence microscopy. As depicted in **Fig 8.**, the functional presence of any of these three multidrug transporters is capable of reducing PG accumulation, probably by extruding PGD (see[16,17,31]), and potentially also PG. In order to estimate the extrusion capacity of the various ABC multidrug transporter for free PG, we performed experiments, shown in **S6 Fig**, by directly measuring PG efflux from preloaded cells, expressing the ABC transporters. As indicated, we have not observed a significant decrease in the cellular fluorescence after replacing the PG-loaded ABCB1 expressing cells into PGD-free media. A slow efflux of free PG, inhibited by ABCG2 inhibitor, was observed in the ABCG2 expressing cells, while a significant, inhibitor-sensitive efflux of PG was observed in the ABCC1 expressing cells. Thus the transport of free PG may not be a major contributor in the case of ABCB1 and ABCG2 extrusion, while ABCC1 (similarly as observed earlier for free Calcein, see ref. [16,17,31]) may significantly transport free PG as well. Especially the PGD, and in some cases the PG efflux together provide the basis of the amplification of the dye extrusion effect in the resulting changes in cellular fluorescence, and the high sensitivity of the assay.

Here we document that this assay can be used for the ABCG2, ABCB1 as well as for the ABCC1 drug transporters, providing a new, unique possibility to examine the functional properties of these key human multidrug transporters by using the same reagents and conditions. In addition, we document that low numbers of ABC transporter positive cells can be distinguished and sorted out from mixed cell populations. Moreover, after a short-term PG accumulation the cells do not show a sign of growth change or toxicity. While DNA-reactive fluorescent transporter substrates may cause major genetic alterations, the cytoplasmic PG accumulation does not seem to have such an effect. Therefore, cell sorting and further selective cell culturing can also be supported by this method.

In summary, the PGD uptake and PG accumulation assay, complemented with the use of selective transporter inhibitors, is a new, highly sensitive tool to examine the functional properties of the key multidrug transporters, and to efficiently select and sort transporter-expressing cells.

## Supporting information

S1 Fig Western blot anti-ABCG2 (BXP-21) staining, and flow cytometry anti-ABCG2 (5D3) immunofluorescent staining of ABCG2. **Panel A**. Control PLB cells and ABCG2-expressing PLB cells**Panel B**. Control A431 cells and ABCG2-expressing A431 cells
(TIF)

S2 Fig Western blot anti-ABCB1 (C219) staining, and flow cytometry anti-ABCB1 (MRK16) immunofluorescent staining of ABCB1. **Panel A**. Control PLB cells and ABCB1-expressing PLB cells**Panel B**. Control A431 cells and ABCB1-expressing A431 cells
(TIF)

S3 Fig Western blot anti-ABCC1 (MRPm6) staining, and flow cytometry anti-ABCC1 (QCRL3) immunofluorescent staining of ABCC1. **Panel A**. Control HL60 cells and ABCC1 expressing HL60 cells
**Panel B**. Control HEK cells and ABCC1 expressing HEK cells
(TIF)

S4 Fig Time dependence of Calcein accumulation in human PLB and HL-60 cells, respectively, expressing the ABC multidrug transporters. The cells were incubated with Calcein AM (Ca-AM) for the indicated time periods, to study the potential differences in the cellular esterase activity, and the effects of the ABC transporter inhibitors (see main manuscript).
**Panels A and Panel B**: Calcein accumulation in control, ABCG2 and ABCB1 expressing PLB cells, after the addition of 100 nM (Panel A) or 500 nM (Panel B) Calcein AM, and the inhibitor of ABCG2 (Ko143) or that of ABCB2 (tariquidar, TQ).
**Panels C and Panel D:** Calcein accumulation in the control, and the ABCC1 expressing HL-60 cells, after the addition of 100 nM (Panel C) or 500 nM (Panel D) Calcein AM, and the inhibitor of ABCC1 (benzbromarone, BB).
These experiments show similar cellular esterase activities in the control and the ABC transporter expressing cells, respectively. Benzbromarone significantly inhibits cellular esterase activity, independent of ABC transporter expression.
(TIF)

S5 Fig Time dependence of PhenGreen accumulation in human PLB cells and in PLB cells expressing ABCG2. Effect of the ABCG2 inhibitor Ko143.
**Panel A**: PG accumulation in the presence of 0.5μM PGD
**Panel B:** PG accumulation in the presence of 2.5μM PGD
(TIF)

S6 Fig Time-dependent efflux of free PhenGreen (PG) from human PLB and HL-60 cells, respectively, expressing the ABC multidrug transporters. The cells were pre-incubated with PhenGreen Diacetate (PGD) for 30 min, then the efflux of free PhenGreen (PG) was measured to estimate the potential effects of the transporters on free PG extrusion (see Main manuscript).
**Panels A and Panel B**: PG efflux from control, ABCG2 and ABCB1 expressing PLB cells–effects of the inhibitor of ABCG2 (Ko143) or that of ABCB2 (tariquidar, TQ).
**Panel C:** PG efflux from control and ABCC1 expressing HL-60 cells, effect of the inhibitor of ABCC1 (benzbromarone, BB).
These results indicate that free PG is not extruded by the ABCB1 transporter, there is a measurable, although slow extrusion of free PG by the ABCG2 transporter, while the ABCC1 transporter is involved in a significant extrusion of free PG (see main manuscript).
(TIF)

S7 Fig MDR activity factor measurements based on mitoxantrone (MX) accumulation in various cell lines expressing ABCB1 or ABCC1 transporter. MX accumulation was measured in the indicated cell lines in the presence of 1μM MX, for 60 min at 37°C.
(TIF)

S8 Fig Structure of PhenGreen diacetate and the product after esterase activity. [19].
(TIF)
