# Supplementary figures and images for: A new fluorescent dye accumulation assay for parallel measurements of the ABCG2, ABCB1 and ABCC1 multidrug transporter functions
Source: PLoS One. 2018 Jan 17;13(1):e0190629. doi: 10.1371/journal.pone.0190629 (PMC5771559; doi:10.1371/journal.pone.0190629)

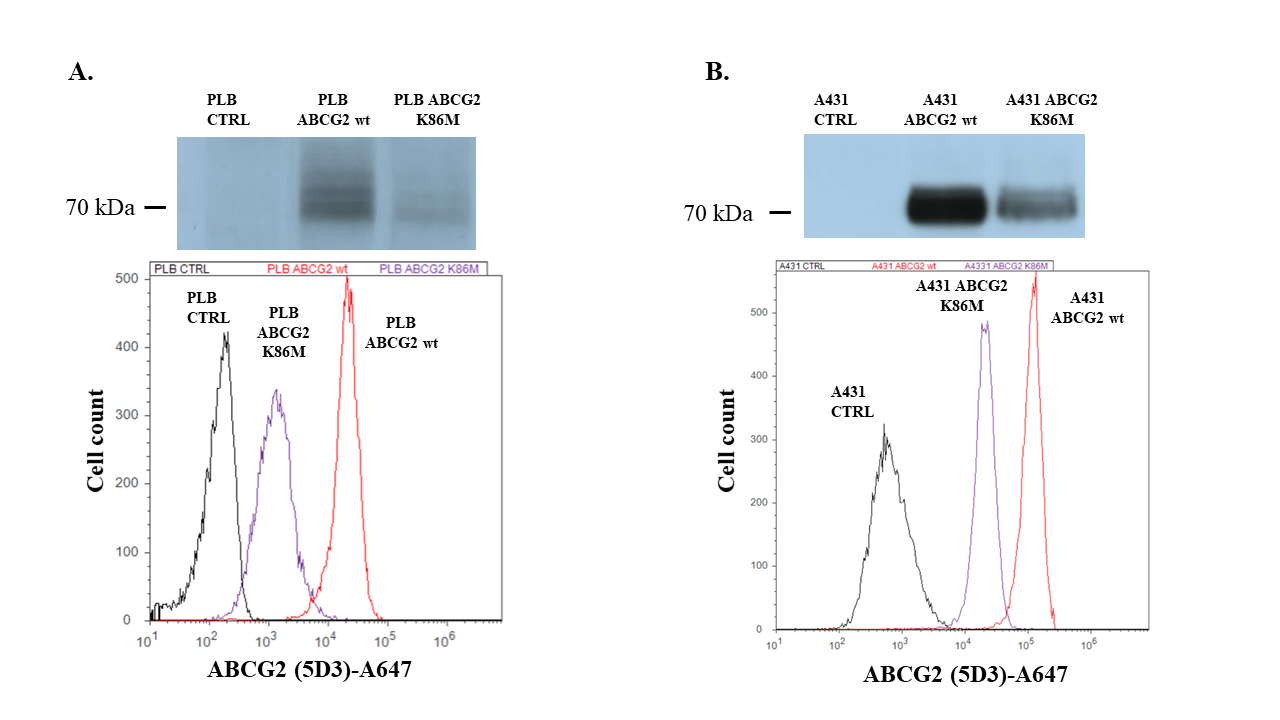

Supplement: S1 Fig — Panel A. Control PLB cells and ABCG2-expressing PLB cellsPanel B. Control A431 cells and ABCG2-expressing A431 cells (TIF) [file pone.0190629.s001.TIF]

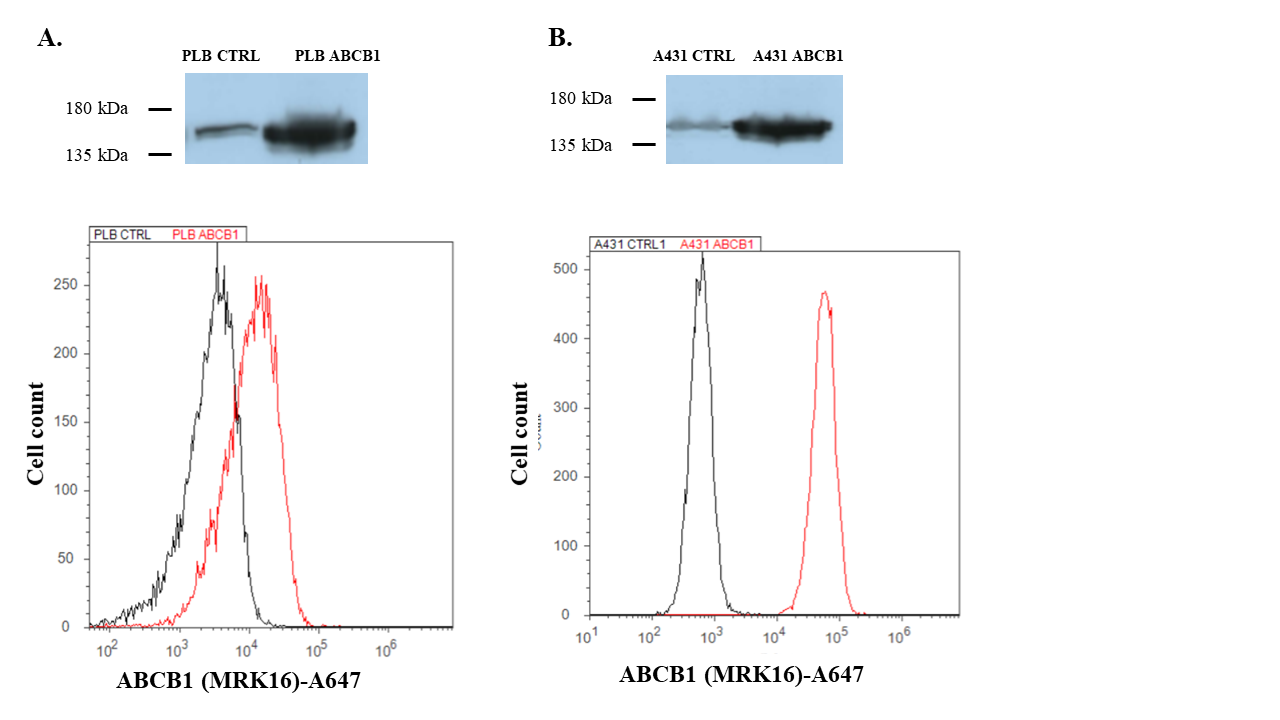

Supplement: S2 Fig — Panel A. Control PLB cells and ABCB1-expressing PLB cellsPanel B. Control A431 cells and ABCB1-expressing A431 cells (TIF) [file pone.0190629.s002.TIF]

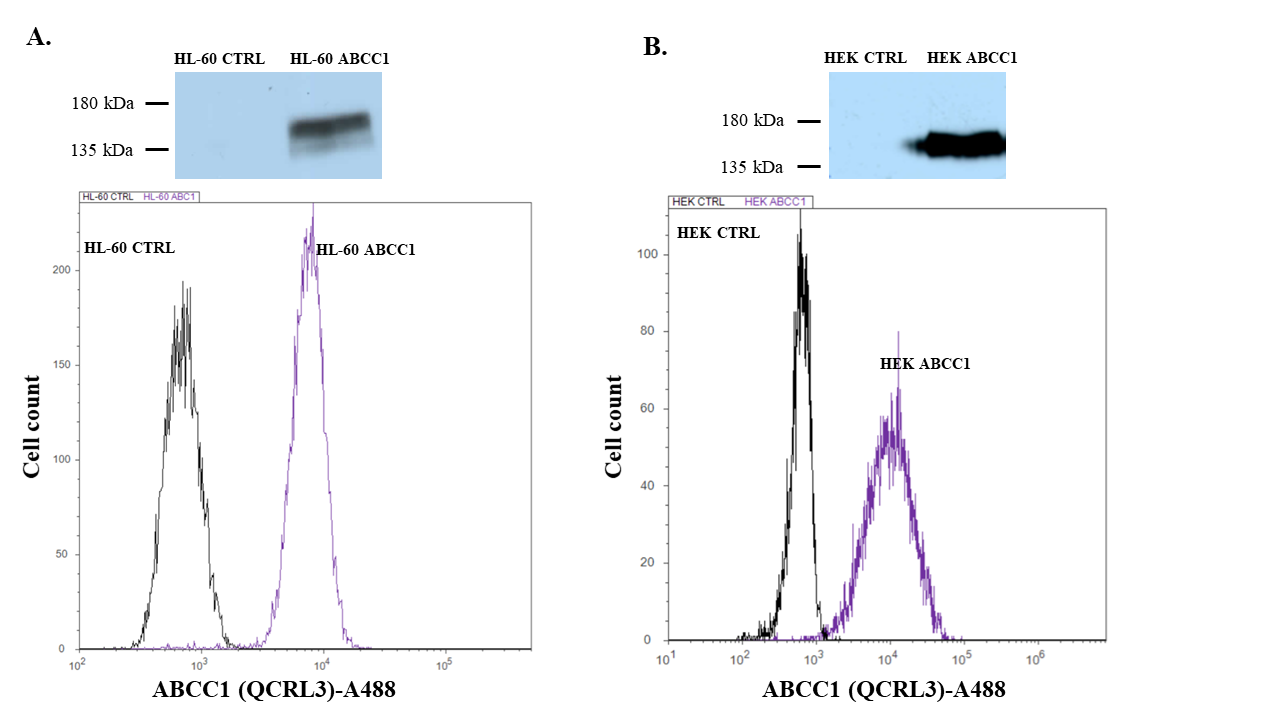

Supplement: S3 Fig — Panel A. Control HL60 cells and ABCC1 expressing HL60 cells Panel B. Control HEK cells and ABCC1 expressing HEK cells (TIF) [file pone.0190629.s003.tif]

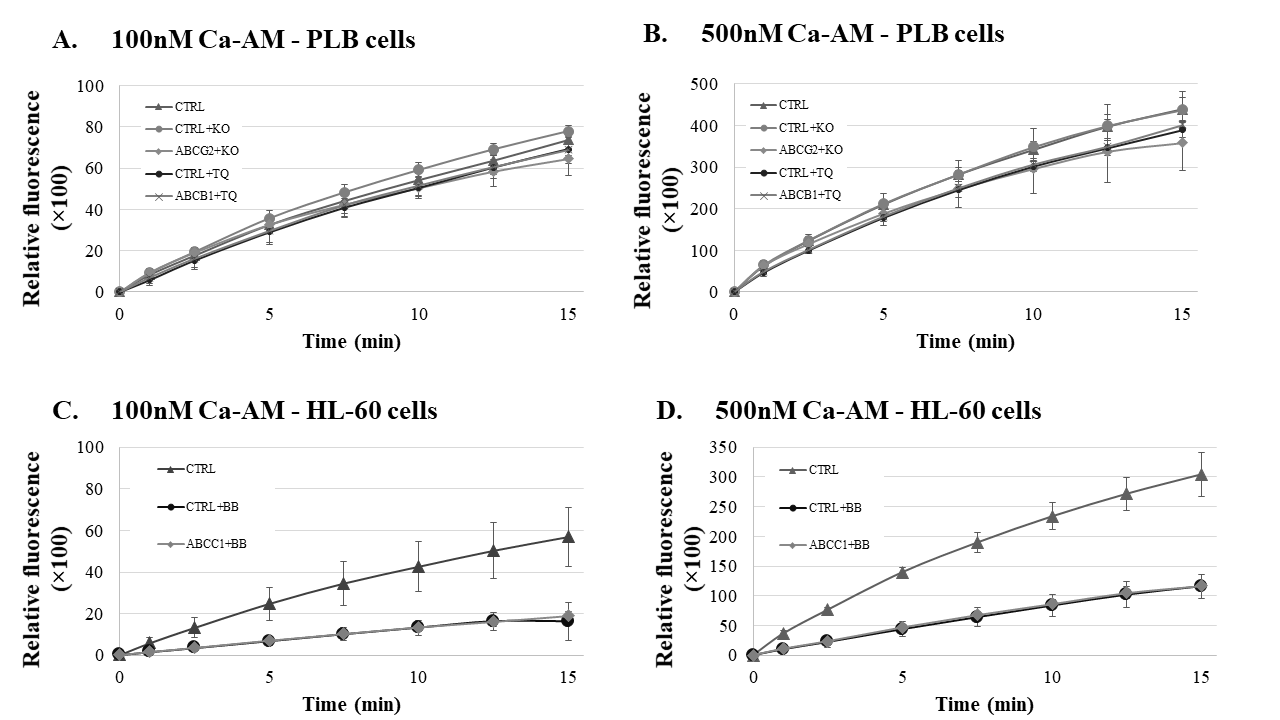

Supplement: S4 Fig — The cells were incubated with Calcein AM (Ca-AM) for the indicated time periods, to study the potential differences in the cellular esterase activity, and the effects of the ABC transporter inhibitors (see main manuscript). Panels A and Panel B: Calcein accumulation in control, ABCG2 and ABCB1 expressing PLB cells, after the addition of 100 nM (Panel A) or 500 nM (Panel B) Calcein AM, and the inhibitor of ABCG2 (Ko143) or that of ABCB2 (tariquidar, TQ). Panels C and Panel D: Calcein accumulation in the control, and the ABCC1 expressing HL-60 cells, after the addition of 100 nM (Panel C) or 500 nM (Panel D) Calcein AM, and the inhibitor of ABCC1 (benzbromarone, BB). These experiments show similar cellular esterase activities in the control and the ABC transporter expressing cells, respectively. Benzbromarone significantly inhibits cellular esterase activity, independent of ABC transporter expression. (TIF) [file pone.0190629.s004.tif]

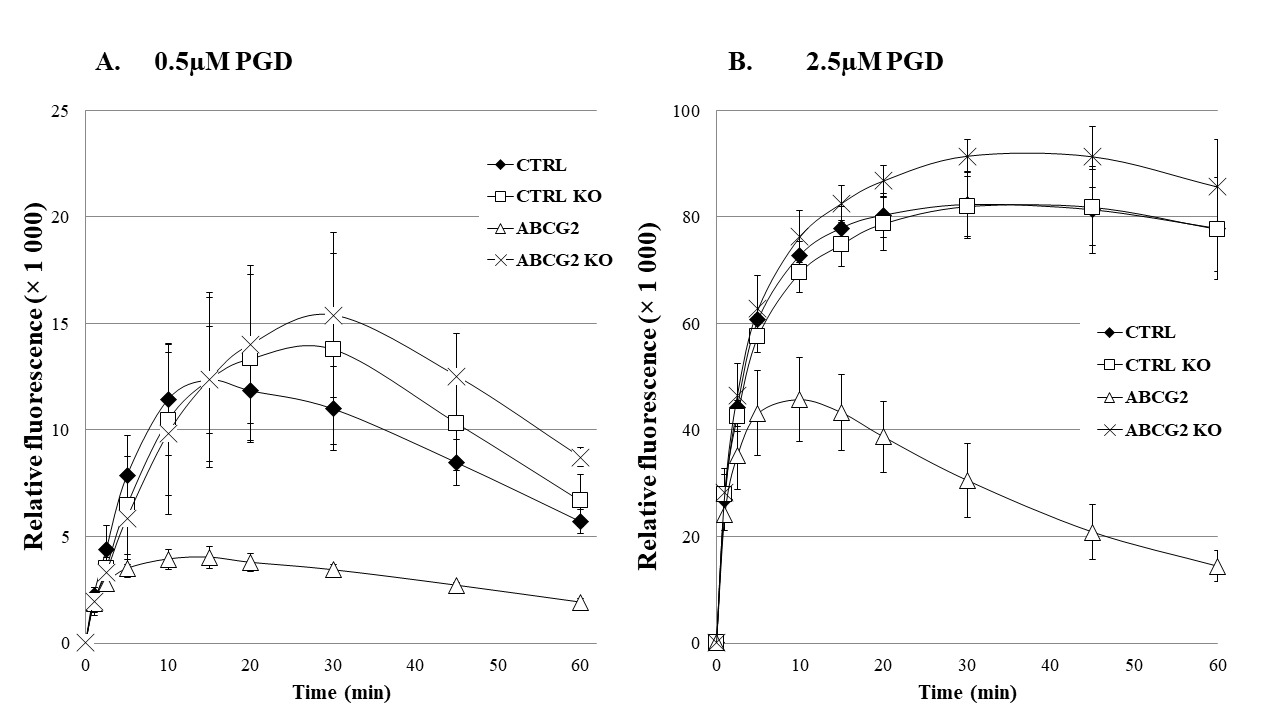

Supplement: S5 Fig — Effect of the ABCG2 inhibitor Ko143. Panel A: PG accumulation in the presence of 0.5μM PGD Panel B: PG accumulation in the presence of 2.5μM PGD (TIF) [file pone.0190629.s005.tif]

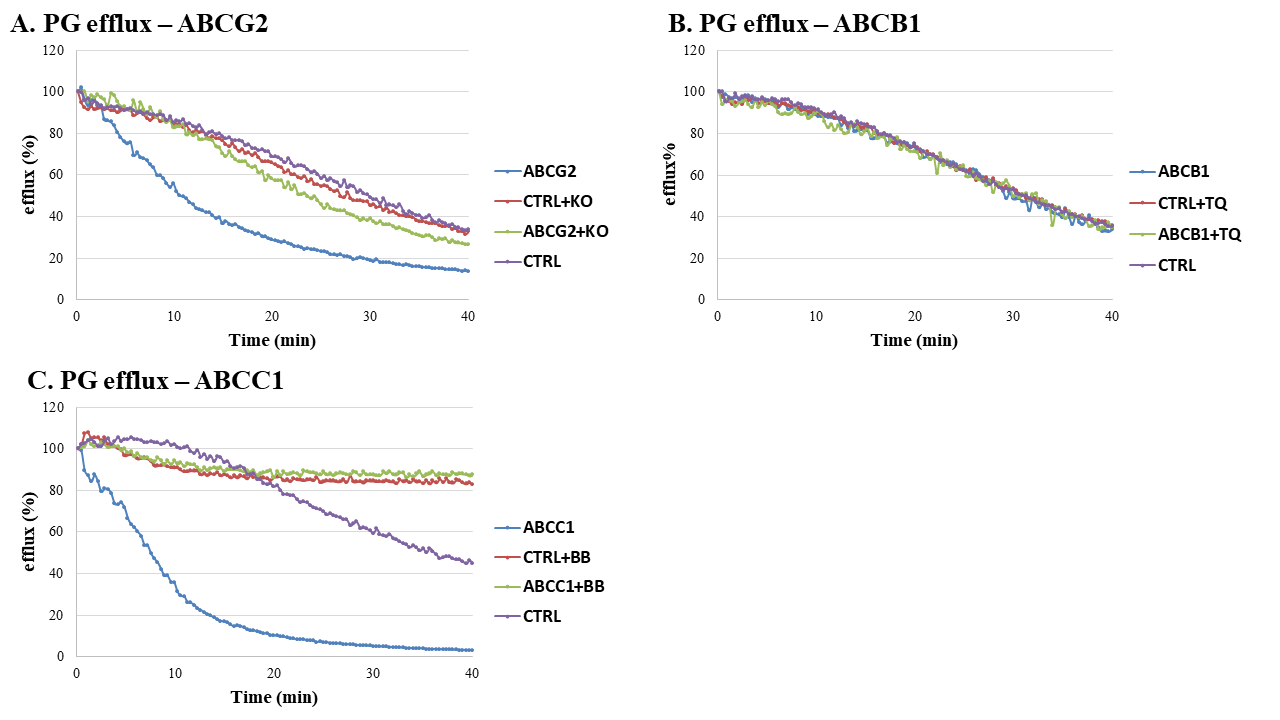

Supplement: S6 Fig — The cells were pre-incubated with PhenGreen Diacetate (PGD) for 30 min, then the efflux of free PhenGreen (PG) was measured to estimate the potential effects of the transporters on free PG extrusion (see Main manuscript). Panels A and Panel B: PG efflux from control, ABCG2 and ABCB1 expressing PLB cells–effects of the inhibitor of ABCG2 (Ko143) or that of ABCB2 (tariquidar, TQ). Panel C: PG efflux from control and ABCC1 expressing HL-60 cells, effect of the inhibitor of ABCC1 (benzbromarone, BB). These results indicate that free PG is not extruded by the ABCB1 transporter, there is a measurable, although slow extrusion of free PG by the ABCG2 transporter, while the ABCC1 transporter is involved in a significant extrusion of free PG (see main manuscript). (TIF) [file pone.0190629.s006.tif]

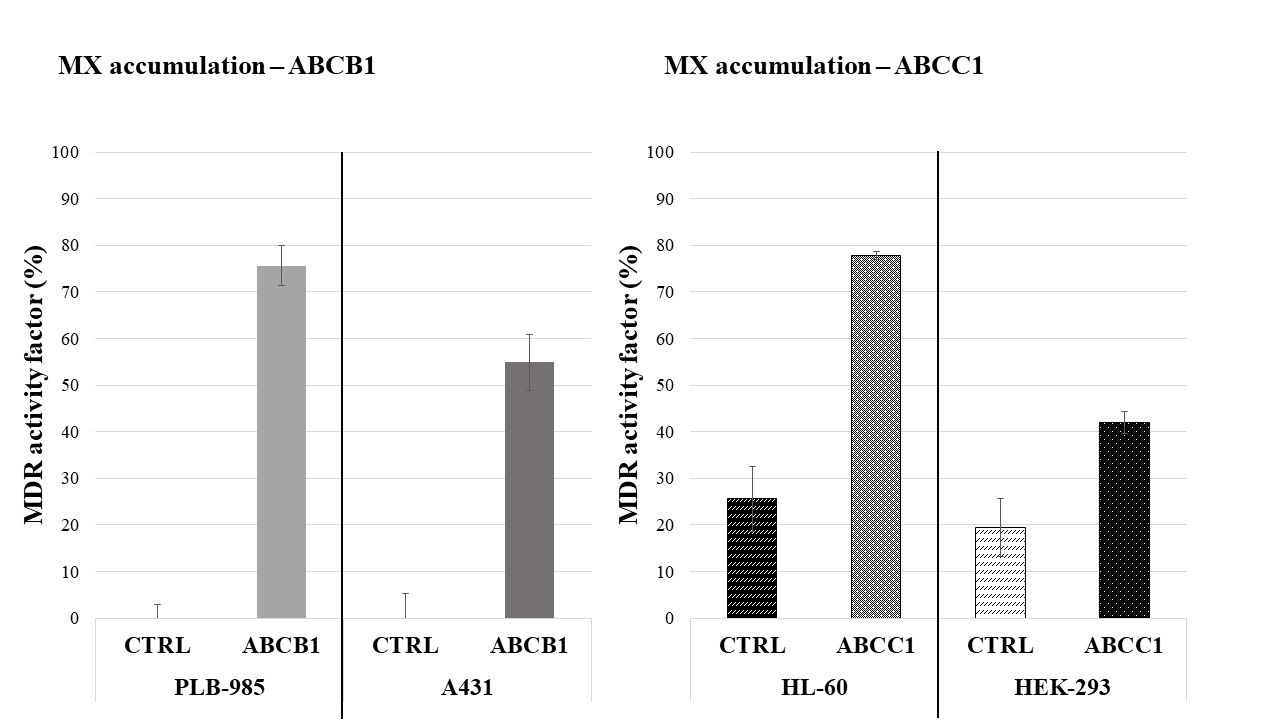

Supplement: S7 Fig — MX accumulation was measured in the indicated cell lines in the presence of 1μM MX, for 60 min at 37°C. (TIF) [file pone.0190629.s007.tif]

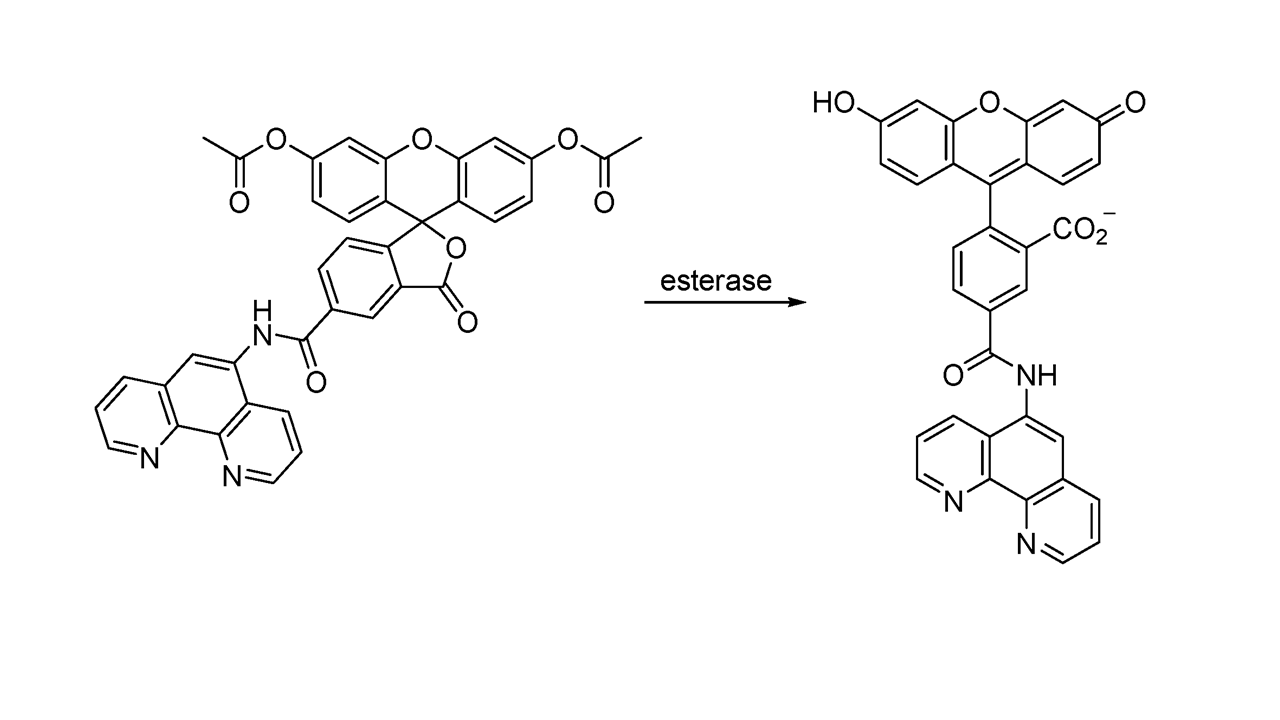

Supplement: S8 Fig — [19]. (TIF) [file pone.0190629.s008.tif]
